# BCMA-Directed CAR T-Cell Therapy in Patients with Relapsed/Refractory Multiple Myeloma and Renal Impairment

**DOI:** 10.3390/curroncol33020080

**Published:** 2026-01-30

**Authors:** Alma Habib, Nausheen Ahmed, Abdullah Mohammad Khan, Darryl Chang, Barry Paul, Hira Shaikh, Christopher Strouse, Emily Struble, Andrew Vegel, Zahra Mahmoudjafari, Muhammad Umair Mushtaq, Joseph P. McGuirk, Al-Ola Abdallah, Shebli Atrash, Reed Friend

**Affiliations:** 1 Division of Hematologic Malignancies & Cellular Therapeutics, University of Kansas Medical Center, Kansas City, KS 66160, USA; 2 US Myeloma Innovations Research Collaborative (USMIRC), Kansas City, KS 66103, USA; 3 Division of Hematology, The Ohio State University, Columbus, OH 43210, USA; 4 Atrium Health Levine Cancer Institute, Wake Forest University School of Medicine, Charlotte, NC 28204, USA; 5 Division of Hematology, Oncology, and Blood & Marrow Transplantation, University of Iowa, Iowa City, IA 52242, USA

**Keywords:** multiple myeloma, chimeric antigen receptor T-cell therapy, BCMA, renal failure

## Abstract

The pivotal clinical trials, CARTITUDE-1 and KarMMa-3, showed promising response rates in relapsed and refractory multiple myeloma (RRMM) with use of BCMA-directed CAR T-cell therapy; however, a major challenge is determining suitability in patients who do not meet trial inclusion criteria due to suboptimal organ function. In this multicenter retrospective study, we evaluated the safety and efficacy of BCMA CAR-T therapy in patients with RRMM and renal impairment (RI), defined as creatinine clearance (CrCL) of less than 45 mL/min. We evaluated 223 patients treated with idecabtagene vicleucel (ide-cel) or ciltacabtagene autoleucel (cilta-cel) between May 2021 and April 2024. Outcomes were compared between baseline RI (11.2%) and normal renal function (nRF) cohorts. Response rates were similar at 1 month (*p* = 0.09), 3 months (*p* > 0.9), and 6 months (*p* = 0.8). Progression-free survival (PFS) was 21.9 months in the RI group compared to 15 months in the nRF group (*p* = 0.32), while overall survival (OS) was 27.9 months for patients with nRF versus not reached for patients with RI (*p* = 0.87). Patients with RI had higher rates of immune effector cell-associated neurotoxicity syndrome (ICANS) (60% vs. 19%, *p* = 0.04) and infections (44% vs. 20%, *p* = 0.008). We found that BCMA CAR-T demonstrated comparable efficacy in RRMM patients with baseline RI, although these patients exhibited increased rates of neurotoxicity and infections.

## Simple Summary

Multiple myeloma (MM) is currently an incurable disease. To advance the treatment options for MM, several clinical trials are testing novel therapies; however, individuals with organ dysfunction, such as renal impairment, often are not enrolled due to strict inclusion criteria. In this study, we evaluated the safety and efficacy of B-cell maturation antigen (BCMA) chimeric antigen receptor T-cell therapy (CAR-T) in the treatment of aggressive, relapsed, and refractory MM. We found that BCMA CAR-T has comparable efficacy in patients with renal impairment, although there are potential increased rates of adverse effects of the treatment including neurotoxicity and infections.

## 1. Introduction

Multiple myeloma (MM), a hematologic malignancy characterized by clonal expansion of plasma cells within the bone marrow, causes a spectrum of end-organ dysfunction and remains incurable [1]. Renal failure due to cast nephropathy is a well-known monoclonal complication of MM [2]. Elevated concentrations of monoclonal free light chains (FLC), resulting from the clonal expansion of plasma cells, is associated with tubulointerstitial injury [3]. Prior studies have shown an early reduction in FLC to significantly predict renal recovery and a survival advantage [3]. FLC reduction is also a key predictor for renal recovery identified by the International Myeloma Working Group.

The treatment of MM has advanced significantly over the past decade, including therapies targeting the expression of B-cell maturation antigen (BCMA) in malignant plasma cells that have led to new treatments [4]. BCMA-directed immunotherapies, including chimeric antigen receptor T-cell therapies (CAR-T), bispecific antibodies, and antibody–drug conjugates have revolutionized the treatment of MM, particularly in the relapsed and refractory setting. CAR-T targeting BCMA, such as idecabtagene vicleucel (ide-cel) and ciltacabtagene autoleucel (cilta-cel), received FDA approval following pivotal trials (KarMMa-3 in March 2021 and CARTITUDE-1 in February 2022, respectively) [5,6]. Ide-cel, the first FDA-approved cell-based therapy for MM, is currently indicated for relapsed or refractory multiple myeloma (RRMM) after two prior treatments, while cilta-cel is approved after one line of therapy and lenalidomide refractoriness [7,8].

The CARTITUDE-1 and KarMMa-3 trials showed promising response rates RRMM. However, a key challenge is determining the suitability of BCMA CAR-T for patients who do not meet the trial inclusion criteria due to suboptimal organ function. For example, KarMMa-3 required a baseline creatinine clearance (CrCl) greater than 45 mL/min, whereas CARTITUDE-1 required a minimum of 40 mL/min [6]. Organ function criteria are essential for patient safety in clinical trials, but they can limit the applicability of the results. In this study, we investigated real-world evidence of outcomes and toxicities in patients with baseline renal impairment (RI) receiving BCMA CAR-T for the treatment of RRMM.

## 2. Methods

### Study Population

We conducted a multicenter retrospective study to investigate the use of BCMA CAR-T in patients diagnosed with RRMM who received either cilta-cel or ide-cel at various U.S. academic institutions, in collaboration with the U.S. Myeloma Innovations Research Collaborative (USMIRC). Patients aged 18 years and older who received treatment between May 2021 and April 2024 were included. Lymphodepletion with fludarabine and cyclophosphamide was administered prior to CAR-T infusion in accordance with the manufacturer’s recommendations, and no dose adjustments were made for patients with RI. Patient and disease data were collected by reviewing the electronic medical records under an Institutional Review Board-approved protocol for each participating center. Baseline RI was defined as a CrCl of less than 45 mL/min. This value was selected as it has been utilized as a cutoff in several prior clinical trials evaluating CAR-T therapy. Responses to therapy were evaluated based on the International Myeloma Working Group (IMWG) criteria. Adverse events were graded using the Common Terminology Criteria for Adverse Events (CTCAE v5.0), with specific attention to cytokine release syndrome (CRS) and immune effector cell-associated neurologic syndrome (ICANS). Comparisons were made between patients with baseline RI and those with normal renal function (nRF) prior to BCMA CAR-T. Descriptive analysis was conducted using R software (R version 4.4.2) to summarize continuous variables and dichotomized factors, while Fisher’s exact test and Wilcoxon rank-sum test were used for statistical analyses. Progression-free survival (PFS) and overall survival (OS) were calculated from the time of treatment initiation using a log-rank test with the Kaplan–Meier method.

## 3. Results

### 3.1. Patient and Disease Characteristics

Our cohort analysis included 223 patients who received ide-cel or cilta-cel for the treatment of RRMM. Patient, disease, and clinical characteristics are detailed in Table 1. Twenty-five (11.2%) patients had baseline RI and 198 (88.8%) patients had nRF. None of the patients had end-stage renal disease or required hemodialysis prior to CAR-T. The nRF cohort had a male-to-female ratio of 117:81 (male, 59% vs. female, 41%), whereas the RI cohort had a ratio of 8:17 (male, 32% vs. female, 68%). The median age was similar between the two groups (65 years for nRF and 67 years for baseline RI, range: 34–84 years; *p* = 0.2). White patients comprised 82% of the nRF cohort versus 60% of the RI cohort, which had a higher prevalence of Black patients (36% vs. 15%). The Eastern Cooperative Oncology Group performance status was predominantly 0–1 in both groups (81.5% vs. 88%). Myeloma paraprotein types showed IgG predominance in the nRF group of 61% compared to 44% in the RI group (*p* = 0.3). Cytogenetic high-risk disease, defined by deletion of 17p, t(4;14), and t(14;16), was present in 40% of the patients with baseline RI and 33.8% of the patients with nRF. Patients with RI were more likely to have Revised International Staging System (R-ISS) stage 3 disease (53% vs. 26%; *p* = 0.03), while there was no difference in the rates of extramedullary disease (EMD). Ide-cel was administered to 84% of the baseline RI cohort and 65% of the nRF cohorts, with the remaining patients receiving cilta-cel (*p* = 0.058).

The median number of lines of therapy prior to BCMA CAR-T infusion was 6 (range: 3–13 lines). There were no significant differences in the rates of prior exposure to bortezomib, carfilzomib, lenalidomide, pomalidomide, and anti-CD38 monoclonal antibodies, as most patients in both groups were at least triple-class-exposed and refractory to these treatments (Table 2). Penta-refractory patients comprised 44% and 29% of the baseline RI and nRF groups, respectively. The number of patients who underwent at least one previous autologous hematopoietic stem cell transplantation (ASCT) was also balanced between the baseline RI and nRF cohorts (72% vs. 84%, *p* = 0.2). Of our total cohort, 12.6% received previous BCMA-directed treatment (Table 2), and would have been excluded in the prior clinical trials. Prior BCMA-directed treatments included teclistamab and belantamab mafodotin.

### 3.2. Post-BCMA CAR-T Toxicities and Adverse Events

We evaluated the rates of treatment-emergent adverse events (TEAEs) following BCMA CAR-T infusion in patients with RRMM (Table 3). CRS was nearly universal in both groups, affecting 80% (158/198) of patients with nRF and 96% (24/25) of patients with RI. Severe (grade 3/4) CRS was rare, observed in only 1% (2/198) of patients in the nRF group (*p* = 0.055). The incidence of ICANS was higher in the RI cohort (60%, 15/25) than the nRF cohort (19%, 37/198), with a notable increase in grade 3/4 events (12% vs. 2%; *p* = 0.44). One patient with baseline RI developed grade 5 ICANS. Hospitalization rates due to severe infection differed significantly (*p* = 0.008), impacting 44% (11/25) of the RI cohort vs. 20% (40/198) of the nRF cohort. Hematologic toxicities, including neutropenia, anemia, and thrombocytopenia, were common across both cohorts, although the RI cohort exhibited higher all-grade incidences (84%, 88%, and 84%, respectively) compared to the nRF cohort (76%, 80%, and 74%, respectively). However, grade 3/4 events remained comparable between groups. In total, 11% of patients with nRF and 16% of those with RI received a stem cell boost (*p* = 0.5). All patients across cohorts were hospitalized for monitoring following the infusion, with no significant difference in duration of hospitalization between the nRF and RI cohorts [10 days (range: 8–15) vs. 10 days (range: 7–15, *p* > 0.9)].

### 3.3. Response Rates and Survival

**Figure 1:**
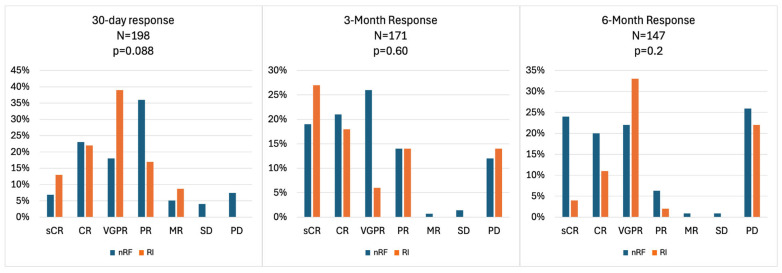
Response rates of patients with normal renal function versus baseline renal impairment (CrCl < 45 mL/min) who received CAR-T for RRMM. nRF: normal renal function, RI: renal impairment.

**Figure 2:**
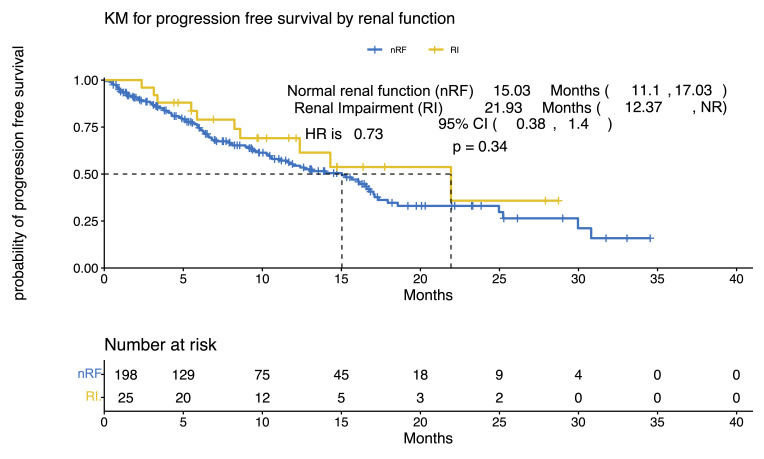
Progression-free survival by renal function in patients who received CAR-T for relapsed/refractory multiple myeloma. The yellow line on the Kaplan–Meier curve depicts patients with baseline renal impairment, and the blue line represents patients with normal renal function. NR: not reached, nRF: normal renal function, RI: renal impairment.

**Figure 3:**
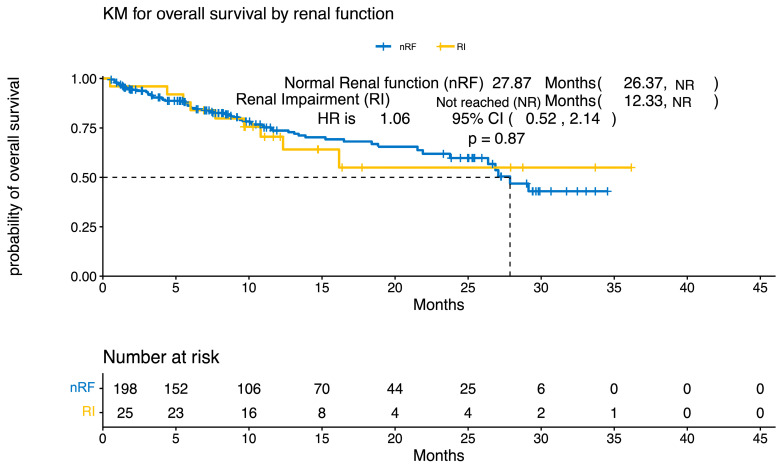
Overall survival by renal function in patients who received CAR-T for relapsed/refractory multiple myeloma. The yellow line on the Kaplan–Meier curve depicts patients with baseline renal impairment, and the blue line represents patients with normal renal function. NR: not reached, nRF: normal renal function, RI: renal impairment.

Both groups showed comparable complete response (CR) rates, with 23% in the nRF cohort and 22% in the RI cohort (Figure 1). At 30 days following infusion, the nRF cohort exhibited a higher partial response (PR) rate of 36% compared to 17% in the RI cohort. However, the RI cohort demonstrated a very good partial response (VGPR) rate of 39%, while the nRF cohort had a rate of 18%. By the three-month mark, the nRF cohort maintained higher rates of CR at 21% and PR at 14%. In contrast, the RI cohort achieved a higher stringent complete response (sCR) rate of 27% compared to 19% in the nRF cohort. At 6 months following infusion, the nRF cohort sustained higher rates of CR at 20% and sCR at 24%, while the RI cohort showed a higher VGPR rate of 33% compared to 22% in the nRF cohort, though this was not statistically significant (*p* = 0.2). Both cohorts achieved significant responses, with the RI cohort showing a deeper response earlier. With a median follow-up range (IQR) of 27.9 months (11; NR), PFS was 21.9 months in the baseline RI cohort versus 15 months in nRF cohort (*p* = 0.3188) (Figure 2). OS was 27.9 months in the nRF cohort versus Not Reached (NR) in the baseline RI cohort (*p* = 0.8715) (Figure 3).

## 4. Discussion

BCMA-directed CAR-T has demonstrated remarkable efficacy in patients with RRMM, including those with baseline RI. Clinical trials for ide-cel and cilta-cel have reported overall response rates (ORR) ranging from 73% to 96.9%, with CR rates between 33% and 67.9% [9,10,11]. In triple-class exposed RRMM, the KarMMa-3 trial demonstrated that ide-cel significantly improved PFS compared to standard regimens, with a median PFS of 13.8 months versus 4.4 months for standard regimens [11]. Similarly, the CARTITUDE-4 trial showed that cilta-cel reduced the risk of progression or death by 74% compared to the standard of care, with a median PFS that had not been reached at the time of the data cutoff [12].

In our real-world cohort, we evaluated the response rates of BCMA CAR-T in patients with RRMM and compared those with nRF (*n* = 198) to those with baseline RI (*n* = 25) at 30 days, 3 months, and 6 months post-treatment. At 30 days, the ORR was not significantly different (*p* = 0.088) between the groups, with CR rates of 23% (40/175) in the nRF cohort versus 22% (5/23) in the RI cohort. At 3 months (*p* > 0.9) and 6 months (*p* = 0.8), the response rates remained statistically comparable across cohorts, with CR rates of 21% (30/140) versus 18% (4/22) at 3 months, and 20% (22/112) versus 11% (2/18) at 6 months, and VGPR rates of 26% (37/140) versus 27% (6/22) at 3 months, and 22% (25/112) versus 33% (6/18) at 6 months, for the nRF versus RI cohorts, respectively. This suggests that BCMA CAR-T has comparable efficacy across different renal function statuses, although there are some differences in the response kinetics. The lack of significant differences also suggests that RI does not markedly influence response durability up to 6 months post-CAR-T therapy. However, the smaller sample size in the RI cohort and higher rates of unknown responses underscore the need for larger prospective studies to validate these findings and assess long-term outcomes to better understand the differences in OS and CR rates in this population compared to the registrational studies.

BCMA CAR-T may contribute to renal recovery in some patients by rapidly reducing tumor burden and FLC levels. For instance, a study of 39 patients with biopsy-proven myeloma of the kidney found that a 60% reduction in FLC by day 21 post-treatment was associated with renal recovery in 80% of the patients [3]. In patients with RI, the incidence and severity of CRS and ICANS appeared similar to those observed in the general RRMM population. However, acute kidney injury after CAR-T cell infusion has been reported in 29% of patients, with severe acute kidney injury independently associated with worse clinical outcomes, including reduced OS and PFS [13].

In our study, CRS was observed in 80% of patients with nRF and 96% of those with RI, with severe cases being rare. However, ICANS was significantly more prevalent in patients with RI, with all-grade incidence rates of 19% versus 60%, and higher severe case rates (2% vs. 12%). This suggests that while CRS rates are similar, those with renal impairment are at a greater risk for ICANS, warranting closer monitoring. A prior study has also suggested that the incidence of AKI was found to be associated with an increased risk of ICANS, although this association warrants further investigation [14].

Hematologic toxicity and infections are a significant cause of morbidity and mortality in patients undergoing BCMA CAR-T. Although hematologic toxicity rates were similar, our cohort showed a significant difference in terms of severe infection, which was higher in the RI than in the nRF cohort (44% vs. 20%, *p* = 0.008), underscoring a potential vulnerability in patients with compromised renal function. Patients with RI may be at a higher risk of infection due to immune dysfunction and the need for prolonged hospitalization. Infections are common complications following BCMA CAR-T, affecting approximately 31–56% of patients, with a mortality rate of 9–15% primarily due to fungal infections [15,16,17,18]. To reduce infection risk, antimicrobial prophylaxis including the use of antivirals and antifungals, is essential. Early detection and prompt treatment of infections are crucial, with hospitalization and broad-spectrum therapies for severe cases recommended [16,17]. Tools such as the CAR-HEMATOTOX score can help risk-stratify patients for tailored prophylaxis and monitoring of hematotoxicity [18,19]. Supportive care, such as growth factor support, CD34+ stem cell boost, and immunoglobulin replacement, can also improve outcomes and reduce infection risk [20]. Our study showed high utilization of CD34+ stem cell boost in RRMM patients treated with BCMA CAR-T.

This study has several limitations. First, its retrospective design may introduce bias due to incomplete documentation of adverse events and outcomes. For example, evaluation of fludarabine-related hematologic and extra-hematologic toxicities and the recovery or deterioration of renal function in patients with baseline RI were not evaluated. Data on the receipt of immunoglobulin replacement at the time of CAR-T infusion was also not available. Variability in practice across centers could impact treatment protocols and reporting standards, potentially confounding results. With the more recent shift to use of cilta-cel, it is important to note that ide-cel predominated over cilta-cel in the study due to the access environment at the time of study coverage. Selection bias may also arise from differing eligibility criteria for CAR-T therapy, skewing the study population toward specific profiles of RRMM patients. Lastly, the study was primarily conducted in academic centers, raising concerns about the generalizability of the findings to community practice settings. Since CAR-T therapy is centralized in specialized facilities at this time, these findings are relevant to real-world contexts; however, additional community-based data could enhance its validation in the future.

## Figures and Tables

**Table 1:** Characteristics of patients with normal renal function versus baseline renal impairment (CrCl < 45 mL/min) who received CAR-T for relapsed/refractory multiple myeloma treatment.

Patient and Disease Characteristics	Normal Renal Function (*n* = 198)	Baseline Renal Impairment (CrCl < 45 mL/min; *n* = 25)	*p*-Value
**Sex, male/female**	117:81	8:17	
**Age, years, median (range)**	65 (34–84)	67 (50–80)	0.2
**Race, no. of patients (%)**			0.043
**Caucasian**	162 (82%)	15 (60%)	
**African American**	30 (15%)	9 (36%)	
**Asian**	2 (1%)	0	
**Hispanic**	1 (0.5%)	1 (4%)	
**Other**	3 (1.5%)	0	
**ECOG**			0.8
**PS: 0–1**	162 (81.5%)	22 (88%)	
**PS: 2**	16 (8%)	3 (12%)	
**PS: 3**	1 (0.5%)	0	
**Unknown**	19 (10%)	0	
**MM paraprotein, no. of patients (%)**			
**IgG**	122 (61%)	11 (44%)	0.3
**Non-IgG**	35 (18%)	7 (28%)	
**Light chain**	41 (21%)	7 (28%)	
**Baseline ISS stage, no. of patients (%)**			0.03
**Stage III**	39 (20%)	1 (4%)	
**Stage II**	74 (37%)	8 (32%)	
**Stage I**	38 (19%)	10 (40%)	
**Unknown**	47 (24%)	6 (24%)	
**Cytogenetics, no. of patients (%)**			
**High-risk disease**			
**Deletion 17 p**	41 (21%)	4 (16%)	0.6
**t(4;14)**	18 (9%)	4 (16%)	0.3
**t(14;16)**	8 (4%)	2 (8%)	0.3
**Extramedullary disease**	75 (38%)	8 (32%)	0.6
**Median no. of previous lines of therapy**	6 (3–13)	6 (3–11)	
**Cilta-Cel**	69 (35%)	4 (16%)	0.058
**Ide-Cel**	129 (65%)	21 (84%)	0.058

**Table 2:** Prior treatment characteristics of patients with normal renal function versus baseline renal impairment (CrCl < 45 mL/min) who received CAR-T for relapsed/refractory multiple myeloma.

Prior Treatment Characteristics	Normal Renal Function (*n* = 198)	Baseline Renal Impairment (CrCl < 45 mL/min; *n* = 25)	*p*-Value ^1^
**Bortezomib exposed**	193 (97%)	25 (100%)	>0.9
**Bortezomib refractory**	106 (54%)	17 (68%)	0.2
**Carfilzomib exposed**	180 (91%)	23 (92%)	>0.9
**Carfilzomib refractory**	139 (70%)	19 (76%)	0.5
**Lenalidomide exposed**	196 (99%)	25 (100%)	>0.9
**Lenalidomide refractory**	152 (77%)	21 (84%)	0.4
**Pomalidomide exposed**	183 (92%)	24 (96%)	>0.9
**Pomalidomide refractory**	150 (76%)	19 (76%)	0.4
**Anti-CD38 monoclonal antibody exposed**	196 (99%)	24 (96%)	0.3
**Anti-CD38 monoclonal antibody refractory**	183 (92%)	23 (84%)	>0.9
**Double refractory**	163(82%)	20 (80%)	0.8
**Triple exposed**	195 (98%)	24 (96%)	0.4
**Triple refractory**	154 (78%)	20 (80%)	>0.9
**Penta exposed**	161 (81%)	21 (84%)	0.3
**Penta refractory**	58 (29%)	11(44%)	0.3
**Number of patients with prior ASCT**	167 (84%)	18 (72%)	0.2
Prior BCMA-directed therapies *	25 (13%)	3 (12%)	>0.9

* Prior BCMA-directed therapies include teclistamab and belantamab mafodotin. ^1^ Fisher’s exact test; Pearson’s Chi-squared test.

**Table 3:** Most common treatment-emergent adverse events in patients with normal renal function versus baseline renal impairment (CrCl < 45 mL/min) who received CAR-T for relapsed/refractory multiple myeloma.

	Normal Renal Function (*n* = 198)		Baseline Renal Impairment (CrCl < 45 mL/min; *n* = 25)		*p*-Value
	All Grades	Grade 3/4	All Grades	Grade 3/4	
CRS	158 (80%)	2 (1%)	24 (96%)	0	0.055
ICANS	37 (19%)	4 (2%)	15 (60%)	3 (12%) *	0.044
Parkinsonism	1 (0.5%)	0	0	0	>0.9
Infection	40 (20%)		11 (44%)		0.008
Neutropenia	151 (76%)	101 (51%)	21 (84%)	14 (56%)	
Anemia	158 (80%)	46 (23%)	22 (88%)	7 (28%)	
Thrombocytopenia	146 (74%)	95 (48%)	21 (84%)	12 (48%)	

* One patient developed grade 5 ICANS.

## Data Availability

The data generated in this study are available upon request from the corresponding author.
